# Optical imaging of prefrontal cortex hemodynamic response in executive function induced by increased cardiovascular activity

**DOI:** 10.1186/1471-2202-15-S1-P34

**Published:** 2014-07-21

**Authors:** Nicoladie D Tam

**Affiliations:** 1 Dept. of Biological Sciences, University of North Texas, Denton, TX 76203, USA

## 

Recent experimental evidence has suggested that an increase in cardiovascular activity resulted from physical exercise can improve cognitive function [1]. We have demonstrated that a short duration of cardiovascular activity can improve executive function [2]. In order to examine the underlying neurophysiological mechanisms that are related to the improvement in cognitive function, we employed optical imaging of hemodynamic activity as a measure of oxygen consumption and oxygen demand in the prefrontal cortex (PFC), so that we can identify whether the improved cognitive function is associated with an increased oxygen perfusion to the brain. It is well known that the PFC is involved in executive function of decision-making that resolves conflicts. We use the conventional Stroop Test to identify the improvement of executive functions in relation to the increased cardiovascular activity. We hypothesize that the improvement in cognitive activity is contributed in part by the increased oxygen perfusion to the PFC. In order to verify this neurobiological mechanisms underlying the improvement in cognitive performance, we use near-infrared spectroscopy (NIRS) to measure not just the neural activation patterns, but also the oxygen delivery to the cortical tissues vs. oxygen consumption by the tissue. Metabolic activities of neurons (such as neural firings and synaptic activations) are correlated with oxygen consumption (oxygen demand) by the neural tissue. On the other hand, oxygen delivery to the tissue is correlated with the oxygen perfusion to the brain (such as vasodilation), which may or may not related to neural activities or cognitive processing. Thus, it is important to identify whether the improved cognitive functions are related to oxygen delivery and/or oxygen demand. We have demonstrated that optical imaging using NIRS can detect neurohemodynamic responses as well as neural activation and deactivation patterns in the motor cortex [3,4]. We have found that under highly demanding neural processing conditions, oxygen delivery may not keep up with the oxygen demand. This results in a transient reduction of oxygen supply when oxygen extraction by the neural tissue exceeded the available oxygen supply by hemoglobin molecules, as revealed by the hemodynamic response using NIRS. This reduction of oxy-hemoglobin supply to the neural tissue could be misinterpreted as neural deactivation by fMRI (functional magnetic resonance imaging), which only detects deoxy-hemoglobin level, whereas fNIRS (functional NIRS) can detect both oxy- and deoxy-hemoglobin levels. The ability for fNIRS to detect both oxy- and deoxy-hemoglobin allows us to differentiate the difference between neural deactivation (revealed by a decrease in oxygen demand or metabolic rate of the neural tissue) and the reduction of oxygen supply (revealed oxy-hemoglobin level) caused by an increase in oxygen extraction (revealed by deoxy-hemoglobin level). Based on this additional information provided by fNIRS, we recorded similar hemodynamic responses in the PFC in this study. Human subjects are used to perform stationary bicycle exercise to increase the cardiovascular activity. Optical imaging of the prefrontal cortex is used to measure the hemodynamic response before and after exercise using NIRS recordings. The performance of the executive function is measured by the Stroop Test before and after exercise. The results showed that the oxy-hemoglobin delivery in the prefrontal cortex increases with the improvement of executive function by comparing the cognitive performance before and after exercise. The processing speed of resolving conflicts in the Stroop Test is also improved by at least 10% after 30 min of exercise, which is correlated with the hemodynamic response in the PFC. It showed that the increase in oxygen delivery is associated with the improvement in executive function in the PFC. This suggests under an increase in cardiovascular activity can increase the oxygen perfusion to the brain, which can provide an improvement in cognitive processing speed in a highly demanding cognitive task (such as the Stroop Test that requires conflict-resolution processing) that requires a transient demand of oxygen that is not kept up with by the oxygen supply during resting condition (without the extra oxygen perfusion and vasodilation caused by exercise).
